# Challenges of a simplified opt-out consent process in a neonatal randomised controlled trial: qualitative study of parents’ and health professionals’ views and experiences

**DOI:** 10.1136/archdischild-2020-319545

**Published:** 2020-11-02

**Authors:** Jenny McLeish, Fiona Alderdice, Helen Robberts, Christina Cole, Jon Dorling, Chris Gale, Members of the WHEAT trial development group, J McLeish

**Affiliations:** 1 Nuffield Department of Population Health, National Perinatal Epidemiology Unit, Oxford, Oxfordshire, UK; 2 Bliss, Maya House, London, UK; 3 Division of Neonatal–Perinatal Medicine, Faculty of Medicine, Dalhousie University, Halifax, Nova Scotia, Canada; 4 Academic Neonatal Medicine, School of Public Health, Faculty of Medicine, Imperial College London, London, UK

**Keywords:** ethics, neonatology, qualitative research

## Abstract

**Background:**

More effective recruitment strategies like alternative approaches to consent are needed to facilitate adequately powered trials. Witholding Enteral feeds Around Transfusion was a multicentre, randomised, pilot trial that compared withholding and continuing feeds around transfusion. The primary clinical outcome was necrotising enterocolitis. The trial used simplified opt-out consent with concise parent information and no consent form.

**Objective:**

To explore the views and experiences of parents and health professionals on the acceptability and feasibility of opt-out consent in randomised comparative effectiveness trials.

**Methods:**

A qualitative, descriptive interview-based study nested within a randomised trial. Semistructured interview transcripts were analysed using inductive thematic analysis.

**Setting:**

Eleven neonatal units in England.

**Participants:**

Eleven parents and ten health professionals with experience of simplified consent.

**Results:**

Five themes emerged: ‘opt-out consent operationalised as verbal opt-in consent’, ‘opt-out consent normalises participation while preserving parental choice’, ‘opt-out consent as an ongoing process of informed choice’, ‘consent without a consent form’ and ‘choosing to opt out of a comparative effectiveness trial’, with two subthemes: ‘wanting “normal care”’ and ‘a belief that feeding is better’.

**Conclusion:**

Introducing a novel form of consent proved challenging in practice. The principle of a simplified, opt-out approach to consent was generally considered feasible and acceptable by health professionals for a neonatal comparative effectiveness trial. The priority for parents was having the right to decide about trial participation, and they did not see opt-out consent as undermining this. Describing a study as ‘opt-out’ can help to normalise participation and emphasise that parents can withdraw consent.

**What is already known on this topic?**

- Much neonatal and paediatric care is not based on robust evidence as definitive randomised trials are lacking.
- Effective and efficient recruitment strategies, such as opt-out consent, have been proposed as a way to increase participation and make trials more generalisable.
- Little is known about parents’ and health professionals’ views and experiences of opt-out consent in the context of an individually randomised neonatal trial.

**What this study adds?**

- The principle of opt-out consent was generally considered feasible and acceptable by health professionals for use in a neonatal comparative effectiveness trial, but was challenging to implement.
- Parents prioritised the right to decide about trial participation for their baby, and they did not see the principle of opt-out consent as interfering with this.
- Describing a study as ‘opt-out’ can help to normalise participation and emphasise that consent is an ongoing process.

## Background

High-quality evidence is often lacking in neonatal and paediatric care^1^ because of a paucity of appropriately powered and methodologically robust trials.^2^ Effective recruitment into randomised trials is needed to facilitate definitive studies.^3 4^ Alternative approaches to consent, such as verbal or delayed consent, are effective and acceptable in particular situations.^5–8^ Opt-out consent, where participants are given full information about a trial and enrolled unless they actively opt out, has been proposed as another way to increase participation^9^ and reduce injurious misconception^10^ and selection bias.^11^ Although studies have asked patients and health professionals for their views *in theory* about opt-out consent in an individually randomised trial,^12 13^ little is known about views *in practice*.

Prevention of necrotising enterocolitis (NEC) is a neonatal research priority.^14^ Red-cell transfusions have been identified as a potential risk factor for NEC,^15^ and observational data suggest that withholding feeds around red cell transfusions may reduce NEC risk; this has not been tested in adequately powered trials.^16 17^ Standard practice on some neonatal units in England is to continue feeds during transfusion, while on others feeds are withheld.^18^

The Witholding Enteral feeds Around Transfusion (WHEAT) pilot was a multicentre, randomised pilot trial carried out in 11 neonatal units in England^19^ that enrolled babies born before 30 gestational weeks and evaluated the feasibility of comparing two care pathways, withholding and continuing feeds around transfusion, on the incidence of NEC. A simplified opt-out consent process developed with parents (box 1) was intended. This paper reports a nested qualitative study exploring the views and experiences of parents and health professionals of the consent process.

Box 1 **Witholding Enteral feeds Around Transfusion pilot trial intended opt-out consent process**

- Parents were approached by a neonatal health professional after their baby was admitted to the neonatal unit, usually within the first 48 hours.
- The health professional gave parents a simple two-page information leaflet about the trial and explained the trial verbally.
- Health professionals explained that babies would be randomly assigned to one of two care pathways around any blood transfusions their baby might need, and that the risks of participation were minimal.
- The health professional explained that all eligible babies were in the trial unless parents opted out, and they could opt out at any time by telling any member of staff.
- There was no consent form.
- The health professional recorded on the electronic patient record that parents had received information and had not opted their baby out of the trial.

## Methods

### Design

This was a qualitative descriptive interview-based study.^20^ The ‘low-inference’ design^20^ was chosen because the purpose was to explore participants’ views and experiences of the consent process.

### Recruitment

The criteria for inclusion in the qualitative study were age ≥18 years, a parent whose baby had been recruited into the WHEAT pilot or a health professional involved in recruitment. Sampling for health professionals involved recruiting one from each neonatal unit, nominated by the WHEAT lead clinician. Due to resource constraints, parents were recruited from four neonatal units comprising a single neonatal network. Any parent with recent experience of WHEAT was invited by the local clinical team; recruitment continued until data saturation was reached within a demographically varied sample.^21^ The interviewer (JMcL) was independent of the WHEAT trial team and had no prior contact with participants.

### Data collection

Data were collected through semistructured qualitative interviews from September to November 2019. Participant information and consent forms were emailed to participants in advance; written or oral informed consent was obtained at the beginning of the interview. Interviews were audio-recorded and professionally transcribed. Interview topic guides are available (online supplementary files A and B).

10.1136/archdischild-2020-319545.supp1 Supplementary data

10.1136/archdischild-2020-319545.supp2 Supplementary data

### Data analysis

Health professionals’ and parents’ transcripts were analysed as separate data sets using inductive thematic analysis,^22^ in parallel with ongoing data collection. Transcripts were checked against audio recordings and reread for familiarity. Codes were identified inductively and recorded using NVIVO. Codes were refined and combined as data collection continued, and themes were identified. JMcL analysed all transcripts and FA analysed a subset; codes and themes were discussed and agreed. Constant comparison^23^ was used to reconsider earlier codes and themes in the light of subsequent interviews. Themes from both data sets were integrated into an overall thematic analysis.

## Results

### Participants

Ten health professionals from the 11 sites took part—one doctor worked across two sites. At seven sites, normal practice was to feed during transfusion; at two sites, feeds were withheld; and at two sites, there was no unit policy. Interviews lasted 19–37 min (mean 25.5); nine were by telephone; and one was by face-to-face in the hospital. Participants’ occupations and normal feeding practice at their unit are shown in table 1.

**Table 1:** Health professionals’ occupations and normal feeding practice

Participant identifier	Occupation	Normal feeding practice during transfusion at their neonatal unit
HP01	Neonatal consultant	No policy: doctor decides
HP02	Neonatal consultant	Continue feeds
HP03	Neonatal research nurse	Continue feeds
HP04	Neonatal consultant	Withhold feeds
HP05	Neonatal nurse	Continue feeds
HP06	Neonatal research nurse	Continue feeds
HP07	Paediatric research nurse	No policy: doctor decides
HP08	Neonatal consultant	Withhold feeds
HP09	Neonatal consultant	Continue feeds
HP10	Neonatal consultant	Continue feeds

Eleven parents of nine singletons and two sets of twins took part in telephone interviews lasting 12–23 min (mean 19); 2 other parents who were invited declined to participate. Five parents identified as white–British, three as British–Asian, one as black–African, one as Asian–other and one as white–other; their ages ranged from 22 to 42 years; and occupation group ranged from semi-routine to higher managerial/professional using the National Statistics socioeconomic classification^24^ (24). Seven had babies who were allocated to the continue feeds pathway, three to the withhold feeds pathway, and one could not remember.

### Findings

Five themes were identified relating to opt-out consent and the comparative effectiveness trial. Table 2 shows illustrative quotations.

**Table 2:** Illustrative quotations from participants

Themes and subthemes	Topic	Quotation
Theme 1: opt-out consent operationalised as verbal opt-in consent		
	Verbal consent—professionals	‘You were going backwards and forwards to see if they wanted to do it or not. And sometimes you felt a bit like you were harassing them because you needed to have a decision’. (HP07)
	Verbal consent—parents	‘They gave us I think two days or one day to make the decision … I just had to give verbally confirmation’. (P04)
	Reasons—electronic patient record	‘When you go into [the electronic patient record] to randomise it, it asked if you had consent from parents … So it was difficult to say it was an opt-out’. (HP05)
	Reasons—unfamiliarity	‘We only really have practised consenting for trials, we haven’t really practised opting out, and I think maybe our language is wrong… it needs to be much, much clearer how that conversation works… Is it that we’ll come back to you and ask you, ‘Do you want to opt out,’ which sounds very much like, ‘Do you want to opt in?’ Or is that if families don’t come back to us with an opt-out then they are in?’ (HP04).
	Reasons—ethical concern	‘I would actually find it really challenging … I would be uncomfortable to put everything on(the parents’)responsibility’. (HP09)
Theme 2: opt-out consent normalises participation while preserving parental choice		
	Normalisation	‘Because a lot of the time they just want normal practice, what everyone else is doing … they don’t actually want to make the decision, but they want to feel like they’ve made a decision’. (HP07)
	Opt-out consent does not compromise parents’ right to choose.	‘Unless we are excluding ourselves you will be enrolled… it’s just our choice, isn't it? Somebody’s giving you a choice, you join or you exclude yourself, it’s down to you’. (P04)
	Preferring not to actively choose	‘With everything that was going on it seemed quite insignificant, so we just passed the decision over to the doctors’. (P08)
Theme 3: opt-out consent as an ongoing process of informed choice		
	Same imperative to secure fully informed choice	‘I don’t think just because you’re opting out that’s an excuse to be less informative’. (HP02).
	Parents had enough information.	‘Someone explains the situation … and then [you] make an informed decision about it’. (P10)
	Parents appreciated a concise information leaflet.	*’*The last thing I wanted was to sit down and read a leaflet’. (P02)
	Brief information was proportionate.	‘It’s very simple, everything is really clear … on the other studies because it involved medication … you required more information to make you to decide’. (P04)
	Parents valued explanation from staff.	‘The way they explained it to me, I was comfortable with it … it’s that human interaction beforehand, that explaining that helps’. (P10)
	Parents felt overwhelmed.	‘I was not of sane mind, like we didn’t know if [the baby] was going to live or not, so to then discuss studies when it’s not really about his personal care, you kind of ignore it’. (P11).
	Traumatic context might affect understanding.	‘At lot of it was blurry … I really didn’t comprehend what they were trying to say. I just said ‘yes’ because it was overwhelming and it was a lot’. (P05)
	Professionals confirmed ongoing consent before intervention.	‘I gave them the option that you can opt out anytime … Before we gave a transfusion we are saying that, ‘They are part of the WHEAT trial, are you happy that we are following exactly as the WHEAT trial?’’ (HP10)
	Parents used opt-out consent to defer decision making.	‘I was happy to put him onto the study because I was like, ‘I don’t really need to make this decision for sure until we find out whether he’s actually going to have a blood transfusion’… What made me feel better was that at any point we could say ‘No, we don’t want to do this anymore’. (P01)
Theme 4: consent without a consent form		
	Benefit to professionals	‘The paper trail was great … beautifully easy’. (HP04)
	Benefit to parents	‘From a parental perspective, they don’t have to sign a document which they will feel more responsible for doing’. (HP09)
	Risk to professionals	‘Almost to cover your own back so that in two weeks’ time, ‘Well, they didn’t tell me that’. And they’ve not signed anything, it’s just my word’. (HP06)
	Some parents request paperwork	‘They were saying that we have to get something on paper, like evidence, so I printed twice the enrolment data from (the electronic patient record)’. (HP03)
	Parents satisfied	‘She then explained that it was an opt-out trial and therefore we didn’t need to sign a consent form… I thought it was okay’. (P01)
Theme 5: choosing to opt out of a comparative effectiveness trial		
Wanting ‘normal care’	Normal care understood as care at this unit	‘(Parents say), ‘We will just do whatever you guys are normally practising here’ … because that’s the only hospital that’s known to them’. (HP09)
	Using examples of staff who have experience in other units	‘We say, ‘Where [that nurse] has worked previously, their normal treatment would be not to feed’ … [parents] can see it in front of them, that there are places close by who may not feed, but we feed and other places don’t and other places do’. (HP05)
	Having normal care means not having to take responsibility for a decision	‘Any kind of standard care the baby will receive will take this kind of relief from [parents], that they don’t have to make that decision’. (HP03) ‘I didn’t want to be the one that had made the decision to change what the treatment would have been, because had that then gone wrong and caused a problem, that would have been due to my decision’. (P01)
A belief that feeding is better	Importance and topic of trial not fully understood	‘It was just generally getting an idea of what the outlook is when you’re in the two different groups, and I think she was telling me that in their opinion it was always very similar and that they therefore thought that it wouldn’t make a difference’. (P01)
	Difficult to explain clinical equipoise without undermining confidence	‘It’s always a slightly odd conversation … If I was a parent I’d think, ‘Well haven’t they learned how to feed babies yet?’’ (HP02)
	Health professionals had personal views	‘Feeding and its putative relation to NEC, I don’t believe there’s any connection myself’. (HP02) ‘Not all staff bought into the idea of … stopping feeds over 11 hours … I don’t know whether that came about in their consenting processes as well’. (HP01)
	Parents had intuitive views of feeding during transfusion.	‘Parents say ‘Why would you stop feeds, because feeding is such a natural thing?’’ (HP01).
	Concern about length of time feeds withheld	‘Parents mainly pick up the fact that 12 hours, that’s quite a long period of time’. (HP03)
	Parents concerned about hunger and weight gain	‘Anytime that they’re not fed it stresses us out…Because they’re only born a pound and so every little gram helps’. (P11)
	Some parents would have subsequently opted out if baby was not randomised to their preferred arm	‘If he had been put in the group where he would have had IV fluids then we would have withdrawn him from the study at that point’. (P08)
	Parents encouraged to defer opting out until after randomisation	‘I told them, ‘Don’t worry, we’ll just randomise them and then we will see, if they are in the feeding group anyway it won’t make a difference, [and] they can actually opt out any time’’. (HP10)

#### Opt-out consent operationalised as verbal opt-in consent

Despite the intention to use opt-out consent, many health professionals described how in practice they had sought consent using a verbal opt-in approach. Usually, there were at least two conversations with parents to explain WHEAT and then inquiry about participation. This was reflected in the experiences of parents, 10 of whom said they were given information and then asked to confirm an explicit choice to participate or not, while one could not remember any discussion of WHEAT.

Health professionals described three factors that had shaped this approach: first, consent was actively recorded in the electronic patient record, and they did not feel they could affirm this unless the parents had positively consented. Second, some health professionals did not feel confident explaining opt-out consent despite training, and staff turnover meant not all were trained. Third, one did not feel it was ethical to proceed without opt-in consent.

#### Opt-out consent normalises participation while preserving parental choice

The phrase ‘opting out’ was used in verbal explanations and the information leaflet. This framing was felt by health professionals to normalise participation in the trial, tapping into parents’ desire for normality in an abnormal situation.

Some parents had not noticed the mention of opting out, while others had understood it and found it acceptable as they did not see it as compromising their right to consent. Several parents confirmed health professionals’ belief that opt-out consent could be less stressful, because they preferred not to have to make an active choice.

#### Opt-out consent as an ongoing process of informed choice

Health professionals were clear that their responsibility to ensure parents made an informed decision was not affected by opt-out consent. Most parents felt that they had been given sufficient information and appreciated that the information leaflet was short and proportionate. They particularly valued explanations from staff. All parents described feeling overwhelmed with their situation following very preterm birth; some parents and health professionals said that any consent given in the first days might not be truly informed because of this. Some parents suggested that consent should be revisited later, and some health professionals noted that they actively reconfirmed ongoing consent when a blood transfusion became necessary. This was felt to be particularly important if there was a long period between the original consent and transfusion. Some parents interpreted opting out as emphasising their right to change their mind about trial participation, and some used this to defer substantive decision making until their baby needed a blood transfusion.

#### Consent without a consent form

Most health professionals thought the absence of a paper consent form was positive and made decision making less stressful for parents. Two were concerned this could potentially leave them exposed to complaints if parents did not remember the consent conversation.

Most parents made no comment on the absence of a consent form. One who had experience of clinical trials was surprised but satisfied with the explanation given.

#### Choosing to opt out of a comparative effectiveness trial

The expectation had been that opt-out consent would simplify recruitment. Health professionals reported that some parents declined participation for reasons that might affect any neonatal trial: they were traumatised and unable to engage with research, were opposed to any intervention not of proven benefit, or the person who approached them lacked the necessary communication skills. However, additional reasons for declining related to parents’ understanding of comparative effectiveness and normal care.

##### Wanting ‘normal care’

Health professionals said some parents were not persuaded by the argument that both trial arms represented normal care in England, but rather focused on normal care at the individual unit. Parents may have prioritised the clinical judgement of clinicians they had met over unknown professionals elsewhere.

Wanting local normal care could also be connected with parents not wanting to be responsible for taking a decision with unknown outcomes; this occurred even at a neonatal unit where both arms of the trial were considered normal care.

##### Belief that feeding is better

Another reason for declining was connected to parents’ perceptions of risk and benefit. While parents said they had specifically asked about risks before deciding, no parents mentioned NEC, despite it being the primary outcome of the trial and being clearly discussed in the parent information sheet, which stated ‘We want to know if feeding babies or not feeding babies while they have a blood transfusion has an effect on the number of babies that get NEC’. Instead, most had a general sense that the trial was about improving care and was perhaps not particularly important. Some health professionals described having difficulty explaining uncertainty around feeding during blood transfusions without undermining parents’ confidence. This may have been linked to the fact that despite clinical equipoise, some had clear personal views about the topic of the trial.

Some parents reported basing their decision to opt out on an intuitive sense that feeding was better than not feeding and that WHEAT required an interruption in feeding which could affect weight gain and hunger. All parents interviewed had consented for their babies to be in WHEAT, but some had shared these concerns. Some parents from the ‘continue feeds’ group said that they might have opted out if their baby had been allocated to the ‘withhold feeds’ group, representing another form of deferred decision making.

## Discussion

Methodologically robust trials are essential to advance clinical care. In the neonatal setting, this means asking new parents to process complex information about uncertainty and risk, and to make consent decisions when their baby is critically unwell. It is perhaps unsurprising that previous studies have questioned the validity of consent given in these circumstances.^25–27^ Despite this, parents overwhelmingly support neonatal research and want to be involved in decisions about their babies’ participation.^8 28 29^ Efficient and effective approaches to consent are therefore key to informed involvement by parents. Opt-out consent in an individually randomised comparative effectiveness trial is novel and is the focus of this study.

Most health professionals believed that opt-out consent was ethically acceptable and advantageous for comparative effectiveness research, and helped to normalise trial participation. This is consistent with patient and health professional support for opt-out consent previously identified in a hypothetical comparative effectiveness trial.^12^ Opt-out consent in WHEAT was, however, operationalised by health professionals as opt-in verbal consent from parents, contrary to the intended process. In part, this was because documentation required active recording of consent, and in part because of what health professionals were used to for clinical trials. Active consent in research contrasts with clinical practice where parents are usually not consulted about comparative effectiveness decisions at all.^30^ As a result of how consent was operationalised by health professionals, this study was not able to explore the views of parents following completely opt-out consent. In keeping with previous work,^9^ concise participant information was acceptable to parents, who found it easy to understand and read. The principle of default participation in comparative effectiveness trials was also acceptable to those parents who understood it. This contrasts with parents’ their views on a hypothetical trial of novel treatments,^31^ but is consistent with views reported in Euricon^26^ and other studies,^9 13^ where what was most important for parents was the right to decide about participation. Parents did not see opt-out consent as infringing on this right.

Most health professionals in this study supported not using written consent forms and this was acceptable to parents, as in the Cord Pilot Trial.^32^ It has, however, been shown in neonatal trials that even when using conventional, written, opt-in consent a proportion of parents do not recollect having consented.^25^ This risk might be amplified by the absence of signed paperwork verifying consent. This troubled some health professionals who felt it might leave them vulnerable to complaints, echoing concerns raised about verbal opt-in consent in perinatal trials.^27 33^ It is not clear whether absence of paperwork in an opt-out trial could have adverse effects on the emotional well-being or trust of parents who did not remember consenting, over and above those that may exist in neonatal trials generally.

The description of the trial as ‘opt-out’ was felt to emphasise that the consent process was ongoing and included the right to change a decision to participate at any time, which has not always been well understood by parents in neonatal trials.^26 29^ Health professionals checked ongoing consent by reminding parents about their right to opt out. This was empowering for parents, several of whom used it to defer a substantive decision until they knew whether their baby needed a blood transfusion, or knew to which trial arm their baby had been randomised. This could result in differential opt-out rates by intervention arm and hence selection bias, impacting the validity of trial findings. Future trials using opt-out consent or delays between consent and the trial intervention should ensure such potential bias is minimised and clearly reported to quantify the magnitude of this potential problem. It is important to highlight that consent for any research should be an ongoing process rather than a one-off event.

This study illustrates the challenges of explaining comparative effectiveness trials, particularly that ‘normal practice’ is based on care across the wider health service rather than an individual unit. It was additionally challenging because parents had intuitive beliefs about the topic of the trial (feeding) and which arm was better for their baby, as is often the case in neonatal and paediatric trials.^32 34 35^ However, unlike parents in the CANDA trial^10^ who had an exaggerated perception of additional risks, parents in WHEAT were not worried about the adverse outcome NEC but were rather focused on the effect of the intervention on their baby being hungry or not gaining weight. Moreover, some health professionals described reservations about the trial, including rejection of equipoise, which may have affected how they communicated the trial to parents. This has implications for training health professionals to explain uncertainty and risk without causing additional anxiety.

### Strengths and limitations

Key strengths of this study are that it included the views and experiences of parents from a wide range of backgrounds randomised into a comparative effectiveness trial, and of health professionals from all trial sites with varied experiences of trial recruitment.

Limitations include that all participating parents had babies in neonatal units where normal practice was to continue enteral feeds during transfusion. Parental reactions from units that withheld feeds or had no standard practice were reported by health professionals, but parents at those units may have had more complex views which would have been missed. A further limitation is that, unlike previous studies,^10 26^ we did not have ethical approval to interview parents who opted out. Instead we describe health professionals’ reports of what those parents had told them. Interviews with parents were carried out up to 12 months after birth, which may have affected parents’ recollections. Finally, bias may have been introduced through reliance on the neonatal units themselves to nominate or invite participation in the qualitative study.

It is important to recognise that this study was undertaken within a comparative effectiveness trial which compared two care pathways that were (and continue to be) standard of care in the UK, and hence, there was considered to be minimal additional risk from randomisation within a trial setting. Therefore, these findings are not necessarily generalisable to trials comparing more high-risk interventions.

## Conclusion

The principle of opt-out consent for a neonatal comparative effectiveness trial was generally considered feasible and acceptable by health professionals. Obtaining opt-out consent was, however, countercultural for some health professionals, and introducing it proved challenging. Beyond the background risks to validity of consent that may affect any neonatal research, no additional risks from the simplified consent approach used in this trial were identified. Parents’ priority was having the right to decide about trial participation. Although parents did not experience a full opt-out consent process, those who understood that the intention was default participation in a comparative effectiveness trial did not see opt-out consent as undermining this right. Describing a study as opt-out can additionally help to normalise participation and emphasise that parents can withdraw consent at any time. Processes should be adapted for opt-out consent, including training health professionals to explain the opt-out process and the concepts of equipoise and comparative effectiveness as they apply to clinical care.

## Data Availability

Data are available upon reasonable request. Following the consent process, the individual qualitative interview transcripts will not be made publicly available.
