# Supplementary material for: Challenges of a simplified opt-out consent process in a neonatal randomised controlled trial: qualitative study of parents’ and health professionals’ views and experiences
Source: Arch Dis Child Fetal Neonatal Ed. 2020 Nov 2;106(3):244–50. doi: 10.1136/archdischild-2020-319545 (PMC8070626; doi:10.1136/archdischild-2020-319545)
Supplement: Supplementary data [file archdischild-2020-319545supp001.pdf]

WHEAT Topic guide for health professionals

Qualitative Study

- **Purpose of research –** identifying what works and what are obstacles to recruitment/ consent to randomisation, and identifying health professionals' views on using a simplified approach to consent.

- **Background –** professional role, level of experience, training for WHEAT, previous experience of clinical trials.

- **Experience of inviting parents to take part in WHEAT**
    - Overall
    - How do you explain the trial to parents? (Could you explain it to me?) (Prompt: benefits/ risks, randomisation)
    - When do you typically initially discuss the WHEAT trial with parents (on the first update in NICU/after a few days/dependent on baby's condition/when blood transfusion is needed)?
    - Do you tailor the information for individual parents? (if so, how – consideration of type of hospital unit)
    - What questions do parents ask?
    - How do parents react to the timing of the invitation?
    - Are you normally involved in the care of the baby anyway, irrespective of the trial (i.e. is there some existing relationship with parents)?
    - Do you see any patterns in who consents/ doesn't consent?
    - What factors do you think affect parents' willingness to participate?
    - In what ways is this similar or different to other trials? (Prompts: 'opt out', timing, etc)

- **Thoughts on 'opt out' process**
    - Ethical? Practical?
    - How do parents feel about this approach? What about compared to a more usual approach (with a signed form and "opt-in").

- **Thoughts on improving recruitment**
    - Do you have any suggestions for improving the language we use to describe WHEAT to parents?
    - Is there anything else we should do differently?

This work is supported by the Medical Research Council (MR/N008405/1)

Experience of Participating in the Wheat Trial: Health professionals Topic Guide
Ancillary study to WHEAT
V1.0 23-May-2019

IRAS ID: 154432 REC Reference: 148/LO/0900 ISRCTN: 62501859
