# Supplementary material for: Challenges of a simplified opt-out consent process in a neonatal randomised controlled trial: qualitative study of parents’ and health professionals’ views and experiences
Source: Arch Dis Child Fetal Neonatal Ed. 2020 Nov 2;106(3):244–50. doi: 10.1136/archdischild-2020-319545 (PMC8070626; doi:10.1136/archdischild-2020-319545)
Supplement: Supplementary data [file archdischild-2020-319545supp002.pdf]

WHEAT Topic guide for parents

Qualitative Study

NB clearly some parents may have little recollection of this, especially if their baby has needed lots of interventions. The topic guide will be used very flexibly according to the situation.

- **Purpose of research –** understanding how parents make decisions about whether to allow their baby to take part in a clinical trial > aim is to improve the process *and* make sure parents have a better experience of being invited to take part in research when their baby is on NICU

- **Background –** age, ethnicity, occupation, partner, time on NICU, baby (gestation at birth, current age, current health), previous experience of clinical trials

- **Experience of being invited to take part in WHEAT**

    - Overall
    - How was WHEAT explained to you? (Prompt: benefits/ risks, randomisation, standard care, opt out)
    - What did you think of the information? (verbal, Patient Information Sheet) (Prompt: easy to understand? Scary?)
    - Did you have any concerns or questions, and how were they dealt with?
    - Can you tell me about what made you decide to let your baby take part?
    - [If with partner] Was the decision made together or by one of you?
    - Who explained WHEAT to you? Were they already involved in your baby's care? Did that make a difference?
    - What did you feel about the timing of the invitation? (& did you have enough time to decide?)
    - What did you feel about the 'opt out' process? (Prompt: how did you understand this?)
    - What impact did being invited to take part in WHEAT have on you?

- **Thoughts on clinical trials in general**
    - What did you know about clinical trials before you were approached?
    - Have you been approached about any clinical trials? (Prompt: did being approached for the WHEAT trial feel similar/different to any other clinical trials)
    - What do you think about randomising people/babies to different treatments in medical research?

McLeish J, *et al. Arch Dis Child Fetal Neonatal Ed* 2021; 106:244–250. doi: 10.1136/archdischild-2020-319545

WHEAT

Qualitative Study

WHEAT Topic guide for parents

- **Thoughts on improving language & process**
  - Do you have any suggestions for improving the language we use to describe WHEAT to parents? [Prompt: specific words/phrases from Patient Information Sheet]
  - Is there anything else we should do differently?

The topic guide is for internal use only and will not be shown to the parents participating.

This work is supported by the Medical Research Council (MR/N008405/1)

MRC Medical Research Council | Imperial College London | NDAU Neonatal Data Analysis Unit | NPEU Clinical Trials Unit | UNIVERSITY OF OXFORD

McLeish J, *et al. Arch Dis Child Fetal Neonatal Ed* 2021; 106:244–250. doi: 10.1136/archdischild-2020-319545
